# Retaining Points

**Published:** 1878-02

**Authors:** Simeon H. Guilford


### RETAINING POINTS.

BY SIMEON H. GUILFORD, A. M., D. D. S.

It is a remarkable fact, singularly illustrative of the weak ness of human nature and the deficiency of human wisdom that while we give proper attention to the greater details of our labor, we generally overlook or neglect the lesser. This too, in the face of the fact, universally recognised and admitted that the great results which we strive to accomplish are in all cases so largely dependent upon the minutiæ.

The failure of scores of fillings in the human teeth, which we annually see and are compelled to acknowledge, may justly be attributed to many different causes; but there is one cause for such failure, which we seldom see or hear referred to; it is the absence of, or the improper making and filling of retaining points or anchorages. The importance of this subject added to its infrequent mention, has prompted the writing of this article. There are cavities where retaining points are not called for, either to aid in the introduction of the filling or its subsequent retention, as in very small cavities, on exposed surfaces, or in any simple cavities, either large are small where the filling is introduced on the wedging principle by the use of cylinders or non-cohesive foil; but in all compound cavities where cohesive foil is needed, or smaller and simple ones, where they are to be filled with this material, retaining points are absolutely required; and being required, it is of the utmost importance that we obtain the greatest amount of good from their employment.

To do this we must thoroughly understand when and where to make them, and how properly to fill them. A retaining point injudiciously or improperly made will be apt to be more hurtful than helpful to both patient and operater; while one improperly filled is not only of no use, but is a delusion and snare, for we place confidence in it only to be betrayed. In the great majority of instances cavities can be and are so shaped as to retain a filling after it has been introduced, without the aid of an anchorage, but there are occasional cases where the cavities will not admit of being so shaped, and then entire dependence for the retention of the filling must be had upon retaining points rightly placed and properly filled. In the former class of cavities, one retaining point is usually, sufficient whereas in the latter several will be needed. Where but one is indicated, as in proximal cavities in the six anterior teeth superior and inferior, and small proximal cavities in the bicuspids and molars, it should be drilled in the center of the cervical end of the cavity; where two are needed, as in large proximal cavities in the teeth of broader base (bicuspids and molars), they should be drilled in the base, as far apart from one another as possible, and connected together by a narrow groove or canal. Where more are needed, as in the saucer-shaped cavities sometimes found on the masticating or buccal surfaces of molars, they should be placed, if possible, equi-distant from each other and in direction divergent as the feet of a tripod,

In irregular and extensive cavities, where a portion of the teeth has been broken away or requires to be built up, the retaining points should be drilled at such points as, when filled will most effectually secure the filling to the tooth. One's own judgment, cultivated by a failure or two, will be the best guide in such cases.

The best shaped drill for the making of these pits, we have found by experience to be a four-sided one, beveled diagon ally at the point, and in width about one-third the diameter of the shank of an engine bit. A spear pointed drill is objectionable, because the portion of the pit formed by the anterior bevel of the spear, when filled, affords no retention whatever, and a deeper hole is made than is necessary. The hole may be drilled either by means of the engine or by hand but we prefer the latter, because by it we are able to go more slowly, and thus feel our way, and there is less danger of drilling deeper than we intended.

In the drilling two evils, (besides the one just alluded to in regard to depth) are to be guarded against; one is, drilling too near the pulp, and the other, is going too near the edge of the cavity. The former mistake may result in the accidental wound ing of that delicate organ, and the latter is liable to break away a piece of the enamel. To avoid both of these the hole should be drilled entirely in the dentine, close to, but not quite touching, the enamel. The direction of the hole should be, as nearly as possible, parallel to the outside surface of the tooth or root.

The depth of the pit should be just sufficient to cause the drill to catch on the sides on its attempted withdrawal at a slightly different angle from the one at which it entered.

The proper filling of the retaining pit is of the utmost importance, for no matter how judiciously placed or properly made it will be valueless if improperly filled.

Many good fillings have been lost through the imperfect filling of a retaining point. In many cases they are not filled at all, but have only a piece of foil roughly crowded into them —whereas in other cases they are filled with such small pieces of thin, delicate foil, packed perhaps by instruments with coarse serrations, that they possess no strength at all.

Whatever foil is used in filling the pit, one thing is certain they must possess strength. This can be had by the use of heavy foil say No 20, cut into narrow strips, or by any plain foil, possessing strength, folded into a ribbon until its thickness is equivalent to No. 20, or 25. Less than this should rarely be used, and each piece introduced should be no wider than the diameter of the hole.

The best instrument we have found for the introduction of the pieces, is a plugger slightly removed from a straight shape and very small, slightly serrated at the point, and triangular and tapering in the shaft. The small point will carry sufficient foil before it at each introduction, while the tapering, angular shank will, while entering, act as a wedge, and force the pieces firmly against the sides. In this way, also each new piece will find a tapering hole waiting for its reception.

The pieces of foil should not be torn in the process of their introduction, for we want all the natural strength of the gold; but they should be forced and tucked and folded into place with a gradual and firm pressure of hand instrument. One end of each piece should be left slightly protrud ng from the pit, to be folded and carried in along with the next piece introduced. This process should be carried on until the pit is considerably more than full, when the protruding portion should be well malleted down into a solid, compact head.

A retaining point thus made and filled, will, we feel satisfied, be perfectly secure in every way, and will answer all the purposes for which it was intended. It may be built upon without fear of its giving way, and will always remain as an integral portion of the filling.—*Dental Office and Laboratory.*

